# The Development of Spinal Pain Beliefs: Patient Journey Mapping From Onset of Symptoms to MRI: A Qualitative Interview Study

**DOI:** 10.1002/msc.70255

**Published:** 2026-08-07

**Authors:** Christopher Bækhøj Franck, Mathias Frederiksen, Anders Hansen, Tina Junge

**Affiliations:** ^1^ Health Sciences Research Centre UCL University College Odense Denmark; ^2^ Department of Psychology University of Southern Denmark Odense Denmark; ^3^ The Danish Rheumatism Association's Rehabilitation Centre Sano Skælskør Denmark; ^4^ Medical Research Unit Spine Center of Southern Denmark University Hospital of Southern Denmark Kolding Denmark; ^5^ Department of Regional Health Research University of Southern Denmark Odense Denmark

**Keywords:** beliefs, health professionals, Malterud, patient journey mapping, qualitative research, semi‐structured interview, spinal pain

## Abstract

**Background:**

Beliefs about pain influence not only how individuals experience spinal pain but also their management behaviours. Health professionals are considered to play a central role in shaping these beliefs, yet little is known about how they evolve over time and which factors contribute to their development. This study explored how spinal pain beliefs develop across the patient journey from symptom onset to the point of MRI examination, applying the method of Patient Journey Mapping.

**Methods:**

Semi‐structured interviews were conducted with six individuals experiencing long‐term spinal pain. Interviews were transcribed ad verbatim and analysed using Malterud's systematic text condensation. An illustrative patient journey was then mapped, linking final code groups to touchpoints that reflected the development of pain beliefs in one informant.

**Results:**

Three code groups were constructed: (1) Influence of health professionals, (2) Societal and cultural influences, and (3) Lived experiences with pain. Informants' beliefs about spinal pain were dynamic, shaped and reshaped by influences across the three code groups, showing considerable change over time. Encounters with health professionals emerged as particularly influential, often reinforcing biomedical understandings. However, when information was conflicting or incongruent, it frequently gave rise to doubt and uncertainty.

**Conclusion:**

This study demonstrates the value of Patient Journey Mapping in uncovering how spinal pain beliefs dynamically evolve through cumulative influences of healthcare encounters, social context, and lived bodily experiences. The findings highlight the critical role of health professionals, underscoring the need for clear and consistent communication to foster helpful understandings of pain and avoid reinforcing limiting or maladaptive understandings.

**Abbreviations**
HP: health professionals
MRI: magnetic resonance imaging
SP: spinal pain

## 1 Introduction

Spinal pain (SP) is a common problem that most people experience during their lifetime (Hartvigsen et al. 2018; Hoy et al. 2010). For many years, SP treatment has been centred around biomedical approaches (Haldeman 1990; Lederman 2011), accompanied by a steady increase in costs and procedures (Deyo et al. 2009). However, overall disability and functional limitations have been on the rise (Deyo et al. 2009; GBD 2021 Low Back Pain Collaborators 2023). The individual in pain might experience debilitating consequences from SP, such as depression, anxiety, loss of social identity, and function (Bunzli et al. 2013; Falsiroli Maistrello et al. 2022; Froud et al. 2014). As a result of the invisible nature of SP, for which a specific diagnosis is often unattainable (Maher et al. 2017), some individuals embark on a journey to obtain a diagnosis in order to legitimise their symptoms to the outside world (Toye et al. 2017), with Magnetic Resonance Imaging (MRI) being a particularly common pursuit (Chou et al. 2018).

Contrary to the societal understanding of pain as a result of damage or illness (Caneiro et al. 2021), SP cannot solely be explained by biomedical or biomechanical factors, as multifactorial contributors such as beliefs, fear, and socioeconomic status contribute to how the individual experiences and perceives SP (Cholewicki et al. 2019; P. O’Sullivan et al. 2016). Certain pain beliefs may predict both severity and duration of pain, with individuals endorsing biomedical views, equating pain with structural damage, being more likely to seek medical care than those with more neutral beliefs (Caneiro et al. 2021; Darlow 2016). Although SP beliefs develop due to a multitude of factors (Bunzli et al. 2017), and health professionals (HPs) likely constitute the primary influence (Darlow et al. 2013; Lin et al. 2013), few studies have examined how beliefs about SP evolve; therefore, examining how this development occurs over time may assist in understanding how an individual's pain beliefs are formed, potentially creating avenues for therapeutic intervention.

A novel method used to understand individuals' experiences and interactions with or within the healthcare system is Patient Journey Mapping, a person‐centred approach attempting to visually or descriptively map the patient journey (Davies et al. 2023). After all, only the individual has experienced the entirety of the journey, whereas HPs encounter only fragmented parts of the journey for which they are responsible themselves (Ben‐Tovim et al. 2008). In this study, we utilise Patient Journey Mapping to explore the complex and sometimes strenuous process that individuals with SP go through (Ben‐Tovim et al. 2008; Petersen et al. 2020). As far as the authors are aware, no prior research has examined the development of SP beliefs from the onset of symptoms to the point of receiving an MRI. With extensive consequences for society and individuals, uncovering this area is imperative to better comprehend individuals' experiences with SP. Therefore, this study aimed to explore how pain beliefs evolve across the patient journey from symptom onset to the point of undergoing MRI among individuals with SP referred to a specialised spine centre using Patient Journey Mapping.

## 2 Methods

### 2.1 Study Design

This qualitative study employed semi‐structured interviews and Patient Journey Mapping (Davies et al. 2023) to explore how beliefs about SP develop. Due to the lack of reporting guidelines for Patient Journey Mapping research, this study adhered to the Consolidated Criteria for Reporting Qualitative Research (COREQ) (Tong et al. 2007). Utilising an inductive approach, the empirical data guided the theoretical framework, and Malterud's systematic text condensation approach (Malterud 2012) provided structure and helped organise the interview material in the interpretation and analysis of the data.

### 2.2 Setting and Informants

The study was developed in collaboration with HPs at the Spine Centre of Southern Denmark, University Hospital of Southern Denmark, an outpatient hospital department specialising in surgical and non‐surgical patient management. In addition to operating around 1000 patients yearly, they receive 13,000–15,000 new referrals from general practitioners, chiropractors, and hospital departments across the Region of Southern Denmark (population 1,250,000). Referrals include SP persisting for over 3 months with inadequate response to primary care management, while referrals for surgical emergencies, inflammatory spinal disorders, or malignancy are not accepted. Recruitment of informants from the Spine Centre began on 6 September 2022, and the last interview was conducted on 1 December 2022. When informants were consulted at the Spine Centre, they were asked for consent by a physiotherapist to deliver their names and phone numbers to the authors. After providing written permission, potential informants were contacted by Christopher Bækhøj Franck by telephone. During this call, an initial screening of SP history was conducted, and Christopher Bækhøj Franck purposively selected informants to achieve variation in symptom duration, age, sex, and pain distribution across spinal regions. Eligible informants were subsequently scheduled for interviews. Before the interview, informants were informed of the purpose of data collection, how data would be handled and anonymised in the article, and how informants could withdraw from the study at any time.

The target population of adult individuals with persistent SP with or without radiculopathy was recruited purposefully to understand how different individuals perceived SP and what meaning they ascribed to particular events since the onset of their symptoms. The only exclusion criteria were not being fluent in either Danish or English.

A sample size of six informants was recruited (Table 1). A purposive sampling strategy was applied to identify information‐rich informants, with an emphasis on variation in experiences and backgrounds to capture diverse perspectives on the development of SP beliefs. Sample size was guided by considerations of information power (Malterud et al. 2016), aiming to ensure sufficient depth and variation in the data in line with the exploratory qualitative design. As the Patient Journey Mapping approach involved in‐depth biographical interviews generating rich accounts of individual experiences, a smaller sample was expected to provide sufficient analytical depth and was prioritised over pursuing the less clearly defined goal of data saturation. Recruitment ceased after six informants, when recurring contributors to belief development were identified across interviews alongside meaningful variation in individual trajectories and experiences. The final sample size was also consistent with the scope and timeframe of the study and allowed for an in‐depth analysis of each informant's journey. The authors had no relationship with any informants. Four interviews were conducted in a separate room at the Spine Centre, and two in the informant's own home, according to their preference. Five interviews were conducted in Danish, and one in English. Interview length ranged from 30 minutes to 1 hour and 35 minutes (mean 54 minutes). At the time of the interview, five informants had completed MRI and received an explanation of their findings as part of their diagnostic evaluation at the Spine Centre, while one informant (M2) was interviewed prior to his scheduled scan. A follow‐up interview was scheduled but cancelled due to illness. The initial interview was retained in the analysis.

**TABLE 1:** Characteristics of informants.

Informants	W1	W2	W3	M1	M2	M3
Sex	Woman	Woman	Woman	Man	Man	Man
Age	58	52	37	65	70	38
Pain duration	+2 years	+2 years	+8 years	+25 years	+55 years	+6 years
Region of pain	Low back‐ and neck pain	Mid‐back‐ and neck pain	Low back‐ + leg pain	Low back‐ and neck pain	Low back pain	Low back pain
Employment status	Employed (part‐time)	On sick leave	Unemployed	Retired	Retired	Employed (full‐time)

### 2.3 Semi‐Structured Interviews

A semi‐structured interview guide was developed, inspired by Kvale and Brinkmann (2022), and informed by the expertise of researchers and clinicians with clinical and research experience in the field of long‐term SP. The interview guide included three predefined themes (thoughts and beliefs, spinal pain explanations, and expectations regarding prognosis), each explored in relation to important events across the patient journey. Two pilot interviews with individuals with long‐term SP were conducted, which helped refine the interview guide and enhance the approach to documenting the patient journey. As the purpose of these interviews was methodological refinement rather than data collection, the pilot interviews were not included in the analysis.

During interviews, the main focus was on examining thoughts, beliefs, and expectations about SP related to important events (touchpoints) across the journey from the onset of SP to MRI evaluation at the Spine Centre. Informants were told that the study explored this broader patient journey, including how events along the way had influenced their thoughts, beliefs, and expectations. Accordingly, the interview guide was designed to explore the development of beliefs across the journey, with MRI treated as one of several potential touchpoints rather than the primary phenomenon under investigation. The detailed interview guide is provided in the appendix.

The interviews were conducted by two male, last‐year physiotherapy students (Christopher Bækhøj Franck, Mathias Frederiksen) with prior training in conducting qualitative interviews. At the time of the study, both authors were trained within a biopsychosocial framework and had a particular interest in psychologically informed physiotherapy. Based on this background, they anticipated that interactions with HPs would play an important role in shaping SP beliefs. This pre‐understanding was considered throughout data collection and analysis through ongoing reflexive discussions among the research team, and the iterative analysis actively sought to identify perspectives that could challenge or extend this view. The study was conducted under the supervision of the last author.

Christopher Bækhøj Franck served as the primary interviewer, focussing on building rapport and ensuring that all questions related to each theme were covered, while Mathias Frederiksen took notes of important events and used them to construct a visual map of the patient journey. This setup enabled both parties to revisit earlier touchpoints if new information emerged. At the end of the interview, a map of the patient journey, constructed from sticky notes on paper, was reviewed with the informant. This served as a form of member checking and validation, as transcriptions were not returned to informants.

### 2.4 Data Analysis

Interviews were recorded using a Zoom H1n voice recorder. The recordings were stored on an encrypted external hard drive, and the raw data were transcribed ad verbatim in Express Scribe by Mathias Frederiksen to ensure uniformity in the transcribed material. After the completion of the six interviews, data were interpreted and analysed using Systematic text condensation, developed by Malterud, inspired by Giorgi's psychological phenomenological analysis (Malterud 2012). This strategy consists of 4 analysing steps: (1) Total impression—from chaos to themes; (2) Identifying and sorting meaning units—from themes to codes; (3) Condensation—from code to meaning; (4) Synthesising—from condensation to description and concepts (Malterud 2012, 795). Christopher Bækhøj Franck and Mathias Frederiksen first read the transcripts independently and then met to discuss potential themes. Both authors collaborated through the three remaining steps of the analysis. The analytic process of identifying meaning units was iterative, allowing the authors to step back and review the material to secure an adequate description of the development of SP beliefs over time (Malterud 2012). This iterative engagement with the data enabled identification of both shared patterns and meaningful variation across participants, thereby supporting sufficient analytical depth in relation to the study aim. After developing the final code groups, an illustrative patient journey was mapped, linking code groups to touchpoints that showed the development of pain beliefs in one informant.

## 3 Results

The results of this study reflect an interpretation of the informants' narratives about the aetiology of their SP and their experiences with the diagnostic problem‐solving process, supported by selected quotations used illustratively in line with Malterud's (2012) Systematic text condensation.

Six informants (three women) with long‐lasting SP, ranging in duration from 2 to 55 years, were interviewed. The age spans from 37 to 70 years.

Three code groups, each comprising underlying subgroups, were constructed, grounded in the data from the transcribed material (Table 2): (1) Influence of health professionals. (2) Societal and cultural influences. (3) Lived experiences with pain. A fourth section, *the development of spinal pain beliefs*, presents the biographical development of SP beliefs through one informant's patient journey.

**TABLE 2:** Codegroups and corresponding subgroups.

Codegroups	Subgroups
Influence of health professionals	Given explanations, management advice
Societal and cultural influences	Mirroring others, workplace & municipality, the invisibility of pain
Lived experiences with pain	Pain is multifactorial, fear of serious pathology, incongruence with treatment expectations

### 3.1 Influence of Health Professionals

Throughout the journeys of informants, interactions with HPs significantly influenced how they understood and managed their SP, with substantial weight given to the explanation and interpretation of diagnostic imaging, including MRI. At the time being interviewed, all informants attributed their pain to biomedical causes, with degenerative changes and nerve compression in the spine being reported frequently. Some informants recalled receiving information from HPs that they perceived as upsetting, leading them to believe they had severe spine deterioration, as reflected in one informant's reaction to being presented with her MRI findings:

W2 ‘The rheumatologist calls me and says “it looks completely wrong. It looks really bad and everything.” It was completely worn out, and he thought it was touching the spinal cord as well (…) I thought, oh my’.

While an explanation of MRI results could cause significant distress for some, other informants, who were told about the benign nature of their findings, would find the explanation reassuring:

M3 ‘They had said before that there was always something to be found (on an MRI), but nothing really was found, so they were a bit like, “your back looks fine, it looks just like it should be and it's actually really good”. (…) I was just happy that there was nothing wrong with my back, but that it was the muscle pain and that was why’.

Although causal explanations, based on imaging results, were perceived as particularly credible and satisfactory by informants, HPs were able to provide explanations in the absence of imaging, which significantly impacted how informants understood and managed their SP:

M2 ‘They took out that rubber spine, and you could sit and look where they say “it's because these rods stick out here and get pinched” (…) I sit upright right now, but I'd rather sit like this (in a slouch). And that's because, then all the spines’ discs, or at least down there where my problem is, it will make more room for the nerves, so they don't get pinched’.

In this example, the explanation of an anatomy model guides the sitting behaviour of an informant, even though he preferred a slouched sitting posture. Nerve compression was also a meaningful causal explanation for another informant, who was diagnosed with herniated discs and operated on several occasions. This understanding contributed to the creation of meaning about pain in other bodily regions:

M1 ‘I also have a constant headache, but that's because it's pinching on that nerve there (…) It's here from the neck, I think.’

Most informants expressed having received several causal explanations early on in their investigation process, most of which were related to muscles and mobility. After receiving an MRI, older causal explanations seemed to coexist with the ones based on diagnostic imaging results, after which muscle‐related explanations once again informed the ongoing course of treatment and behaviour of informants:

W3 ‘Everybody told me that I have a very weak core that I have to strengthen, for my lower back to support my body (…) My back was too weak to carry my son.’

### 3.2 Societal and Cultural Influences

Social relationships affected informants' perceptions of their SP across their journeys, shaping beliefs both independently and alongside explanations from HPs. Seeing themselves reflected in others with similar conditions played a significant role in shaping causal beliefs and estimating prognosis. Two informants who had family members with comparable conditions perceived their SP as likely hereditary:

W3 ‘She (grandmother) had it and my father had it, and we all have it. Because my father’s sister, real sister, and their sister, she had undergone 3 operations for the back, so it makes sense that to some extent it could be genetic.’

After receiving causal explanations from HPs, most informants expressed a desire to learn more about their condition. Some of the medical terminology used by HPs was difficult to comprehend, leading informants to seek advice from personal networks and through internet searches. After being informed by her physiotherapist that her pain was caused by spondylolysis, an informant sought additional information online and felt reassured:

W1 ‘I find pictures on the internet that look like the ones (the physiotherapist) showed me, so I think, well okay then, other people had this, and there's even a term for it, so it's not uncommon.’

In their respective workplaces, several informants were presented with ergonomic training, devices, and counselling, which made daily work more difficult and reinforced an understanding of a fragile spine with a need for protection:

W1 ‘As a nursery assistant, I know you're not allowed to pick up children, you can't avoid that anyway.’

Being met with understanding by the outside world was crucial for informants, with most identifying the invisible nature of their pain as their biggest challenge in this regard. Several informants expressed how they were afraid of being looked down upon, as others had a difficult time understanding their suffering:

W2 ‘It's actually not easy to see. So, if I had my arm cut off, or a leg or something, then everyone can see, damn it, you're missing your arm, that's a shame, right? Can we help you with something… But back pain. It makes them think you're such a hypochondriac sometimes.’

Before undergoing MRI, several informants shared the experience of not being believed, feeling as though others implied that their pain was self‐inflicted. Correspondingly, one informant described finding it difficult to be taken seriously by her general practitioner without exaggerating her symptoms:

W1 ‘Maybe there’s a difference in how you come down and deliver your medical history, but I’m just not the type who sits down and whine. I feel like what I say ought to be enough, right?’

### 3.3 Lived Experiences With Pain

Informants made sense of their symptoms by comparing current and past experiences with information from HPs and the outside world. When recalling the onset of their symptoms, some informants situated their pain within the broader context of their lives, pointing to multiple contributing factors rather than a single cause. When symptoms emerged during stressful periods, some described the pain as their body's way of signalling distress:

W3 ‘My body was sort of purging in form of that cystitis (…), severe health issues, emotional appeals, and so much stress and medication and stuff that the back pain also came along. (…) If something is not right with my mind, it reflects in my body.’

Episodes of very intense or unexplained pain sometimes shifted informants' previously multifactorial understanding towards fear of serious pathology. In some cases, severe pain was described as leading to breathing difficulties or even loss of consciousness, which in turn fuelled catastrophizing thoughts:

W3 ‘It's so… Scary. Because it almost feels like I'm dying, and I don't want to feel breathless (…) It's the worst feeling, I'm telling you. To be conscious that you are going to be unconscious and you cannot breathe.’

Troubled by the severity of their symptoms and unable to make sense of them, informants sought explanations for these grueling episodes. Receiving a benign explanation from HPs left some informants feeling hopeless and not taken seriously. An informant articulated the incongruence between pain intensity and the harmlessness of the explanation, leading her to believe that she was to blame for her suffering.

Uncertain about the cause of their pain and in need of help to relieve symptoms, informants sought help from HPs, which, for many, was experienced as being very painful. Even when treatments such as massage or exercise provoked intense discomfort, informants were willing to undergo them again, trusting in the HPs' guidance:

M2 ‘(…) when you were lying there with your nose down in the hole (on the treatment table), you thought, no, won't she finish soon, right? Even though she has only been going for a quarter of an hour, the water trickled out of my eyes, because it was something that hurt like hell (…) After all, it's the way to the result.’

Although the pain associated with a treatment intervention was considered part of the process for some, the pain became unbearable for others. With intense pain and lack of progress, some informants lost faith in the efficacy of the treatment and accuracy of their diagnosis:

W3 ‘I’m not going to do any exercises. Bloody physiotherapy did not work. This rehab did not work. What is happening to me?’

Although this informant had undergone several rehabilitation sessions with physiotherapists without achieving pain relief, cessation of treatment led to self‐blame and doubt as to whether she had given exercise a real chance. For others, lack of progress did not necessarily mean a deviation from treatment but instead led to reflection on the treatment they received.

Many years of pain could alter informants' perception of body image and lower the trust in explanations received from HPs, which did not correspond to the experienced symptoms:

M1 ‘I must have a weak body. I've been told that I don't. That's what they're trying to convince me anyway (…) Well, I don't believe it when you've been through it so many times, right?’

### 3.4 The Development of Spinal Pain Beliefs

**FIGURE 1:**
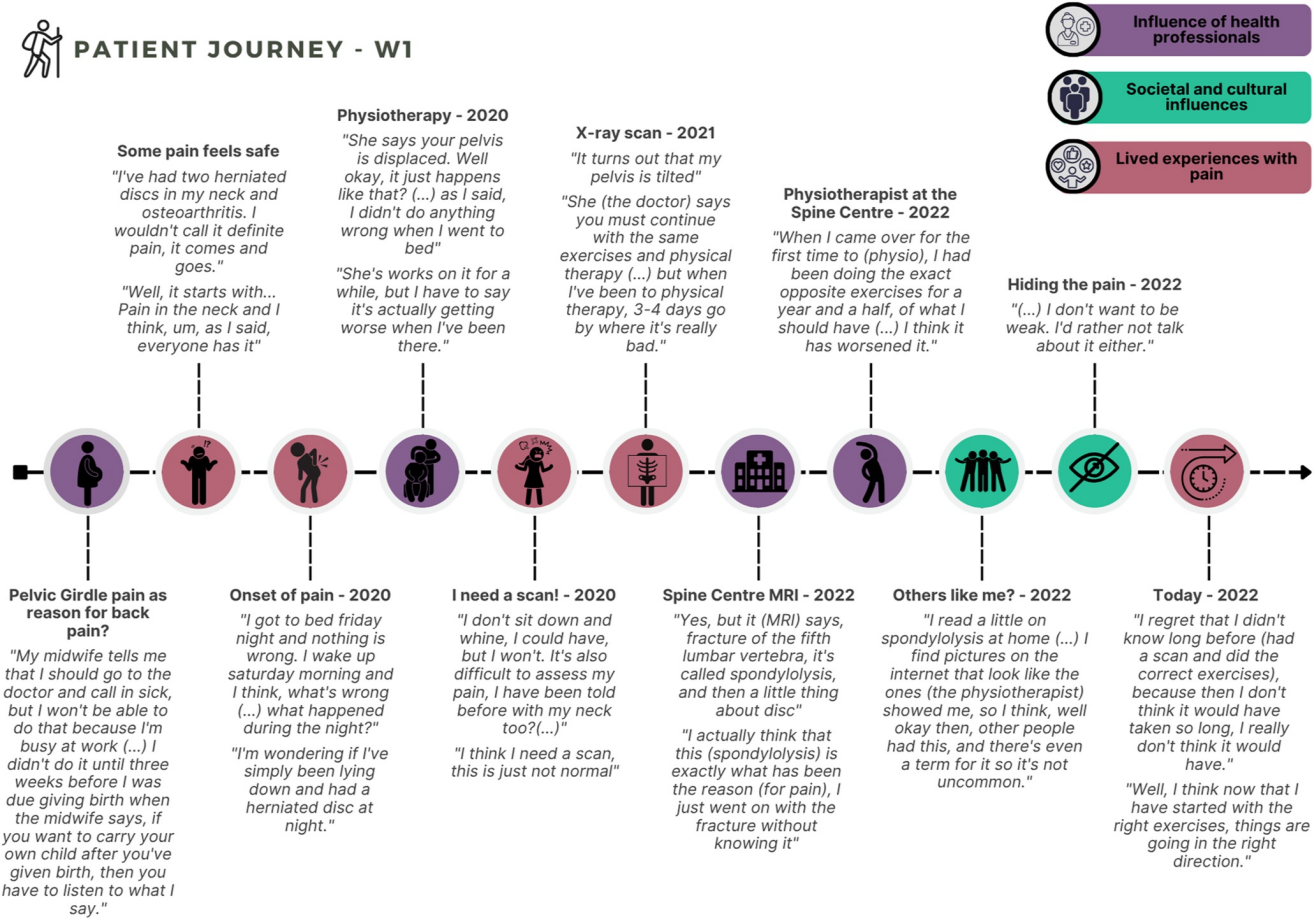
The patient journey of informant W1.

Mapping specific events that have shaped informants' beliefs about pain allows for a deeper understanding of the intricate relationship between personal experiences, interactions with HPs, and society in the formation of SP beliefs. Informants' beliefs evolved continuously, shaped by the ongoing interplay of these three contributors and by whatever information they found most credible, allowing different and sometimes conflicting explanations to co‐exist. Although beliefs developed through similar contributors across informants, the temporal trajectories differed substantially due to variations in pain intensity, duration, and individual patient journeys. Consequently, capturing a single, unified temporal pattern across participants was not feasible. Instead, a representative patient journey was constructed, incorporating elements from all code groups, exemplified by quotes from one informant recalling specific events (Figure 1).

## 4 Discussion

This study explored how SP beliefs evolve in individuals, from the emergence of symptoms through to, and shortly after, undergoing MRI investigation. Based on informants' descriptions, SP beliefs appear to be dynamic rather than fixed, continuously shaped and reshaped over time by influences from within healthcare, society, and lived bodily experiences with symptoms.

Among these, interactions with HPs stood out as particularly impactful, often reinforcing a predominantly biomedical understanding of pain. This finding is consistent with existing literature, highlighting the significant impact of HPs on SP beliefs across diverse cultural contexts (Bonfim et al. 2021; Darlow et al. 2013; Lin et al. 2013; Ray et al. 2022; Riipinen et al. 2022; Setchell et al. 2017; Valenzuela‐Pascual et al. 2021). Through explanations and applications of treatment modalities originating in a biomedical paradigm, HPs may unintentionally reinforce structural and pathoanatomical beliefs that can be detrimental to individuals' action possibilities (Hohenschurz‐Schmidt et al. 2022; Stilwell and Harman 2017; Vaz et al. 2023; Zusman 2013). Such influence may in part reflect challenges in adopting a biopsychosocial model of care (Ng et al. 2021), as HPs with stronger biomedical orientations are more likely to provide treatment and advice that is discordant with existing guidelines (Caneiro et al. 2021; Darlow 2016). Furthermore, the persistence of SP and increase in patient age is often accompanied by a decline in patient‐HP communication quality (Gulbrandsen et al. 2010), which may further explain unhelpful or inadequate understandings of SP originating from healthcare encounters. Individuals' health literacy may also affect how clinical messages about pain are understood (Oosterhaven et al. 2023), sometimes leading to unintended interpretations. Simultaneously, the need to make sense of one's pain and appear credible to others (Toye et al. 2017) may predispose individuals to adopt or emphasise socially acceptable, often biomedical, explanations—especially among those with prior experiences of not being believed (Snelgrove and Liossi 2009). Contrastingly, explanations provided by HPs may also foster reassurance when aligned with current evidence, as illustrated by informants who experienced relief when their MRI findings were explained as benign and consistent with normal age‐related changes.

Some informants expressed multifactorial perceptions of SP at symptom onset. These initial beliefs appeared to be replaced by biomedical causal explanations derived from healthcare interactions early on in the patient journey. This finding contrasts with an Australian Patient Journey Mapping study, which reported that adults hospitalised in the emergency department with acute low back pain already held biomedical beliefs about pain and expectations for diagnostic imaging (El‐Haddad et al. 2018), suggesting that the formation of such beliefs may occur earlier in life. For some individuals, beliefs about pain might develop early in childhood by observing their carers' attitudes and management towards pain (P. B. O’Sullivan et al. 2008).

In this study, some beliefs originated from observing and consulting others as well, where several informants mirrored themselves in close relatives to form an understanding of their condition. As societal beliefs about SP seem to reside in a biomedical understanding of the spine being vulnerable (Darlow 2016; Wand et al. 2023), this might be what informants frequently encounter at both workplaces and when consulting friends and relatives about their pain. Although one informant reported finding reassuring information online, inaccurate and unreliable information is highly prevalent on many internet and social media platforms (Maia et al. 2021; Peterson et al. 2022; van der Noord et al. 2025; Zheluk et al. 2022), highlighting yet another potential avenue for the development of unhelpful beliefs that were not identified in this study.

Beliefs about pain are not only formed through inputs from the outside world (Darlow 2016; Darlow et al. 2015). Lived experiences with pain were decisive for the development of SP beliefs over time among the informants included in this study. If symptoms did not match expectations, it led to doubt and uncertainty about whether the treatment plan and diagnosis were correct. In line with these findings, prior research has found intense pain, uncontrollability, and not understanding symptoms to increase fear and catastrophizing in people with long‐term SP (Bunzli et al. 2015; Ryan and Roberts 2019; Singh et al. 2018). Furthermore, pain affected by light movement or eased by inactivity might create an impression of the back being fragile, whereas experiences with movements that relieve pain might indicate the opposite (Bunzli et al. 2015; Darlow et al. 2015; Stenberg et al. 2014; Wand et al. 2023). Likewise, the duration of symptoms and the scarcity of treatment options appear to affect the beliefs of individuals (Morlion et al. 2021).

Collectively, these results offer an empirical illustration of the biopsychosocial nature of belief formation, highlighting how biological, psychological, and social dimensions are deeply interwoven in shaping individuals' understandings of SP.

Although the clinical implications of SP beliefs in predicting future pain intensity, disability, and management behaviours remain uncertain in the quantitative literature due to limited prospective research (Morton et al. 2019), reductions in pain intensity observed following psychologically informed and exercise‐based interventions are often mediated by constructs reflecting pain beliefs (Alaiti et al. 2022; Schütze et al. 2025), indicating an association between the two. Furthermore, qualitative findings suggest that these beliefs can have enduring consequences for individuals' lives, extending beyond mere understanding of pain to shape behaviours and management strategies (Bonfim et al. 2021; Darlow et al. 2013; Lin et al. 2013; Riipinen et al. 2022). Both the existing literature and the findings of this study emphasise the necessity of explicitly addressing pain beliefs in individuals with SP, ideally prior to initiating treatment. In practice, clinicians can explore patients' prior experiences, treatments, explanations, and current beliefs to identify misconceptions, align understanding, and foster effective, person‐centred care. Unhelpful beliefs can then be addressed through targeted education and behavioural interventions that encourage more accurate and constructive understandings of pain and support self‐management (Caneiro et al. 2021). Considering the substantial influence HPs exert on the formation and reinforcement of such beliefs, insufficient attention to this aspect of care may inadvertently perpetuate maladaptive understandings or give rise to additional, unhelpful explanatory models of pain, potentially causing harm to individuals. As demonstrated in this study, individuals may even endure considerable, and at times unnecessary, discomfort if they are convinced that it will ultimately be beneficial.

### 4.1 Strengths and Limitations

The qualitative interviews generated a detailed dataset. allowing for an explorative examination of pain beliefs among individuals with long‐standing SP through a systematic and rigorous analytic process. The findings offer insight into how such beliefs may develop within a small and selectively referred group of individuals experiencing persistent and often more severe SP than typically seen in primary care (Hansen et al. 2023). This selectivity likely influenced both informants' experiences and the beliefs they expressed and, together with the limited sample size, restricted the breadth of experiences captured. Information on the number of individuals who approached or declined participation was not systematically recorded during recruitment. All informants had a long‐standing history of symptoms (+2 years), providing them with substantial experience with SP and engagement with society. While each patient journey is inherently unique, it is likely that the inclusion of additional participants could have provided further perspectives not captured in the present sample. Nevertheless, this study contributes by identifying shared factors and potential mechanisms that may influence the development of SP beliefs across individuals.

The retrospective mapping of important events represents both a methodological strength and a limitation. While it provided valuable insight into the temporal development of beliefs, it also relied on informants' recollection of thoughts and experiences happening years in the past, introducing the potential for recall bias. Nevertheless, it is plausible that the events in question, often described in vivid detail, were more easily recalled because of their strong emotional significance to informants (Williams et al. 2022).

Although informants' pain beliefs often appeared to originate from explanations provided by HPs, the multifaceted and exact contributions to the formation of their beliefs remain unclear. The informants' narratives reflect their interpretations of interactions with HPs and society and should therefore not be regarded as direct reproductions of those events. Nonetheless, their current understandings of pain and the messages they recall remain highly relevant, given the potential consequences these beliefs may carry for their lives and management strategies. Future observational research should investigate real clinical encounters to better understand how messages from HPs are conveyed, interpreted and ultimately translated into beliefs by individuals with SP.

Patient Journey Mapping offers a valuable approach to exploring the development of pain beliefs across these different influences, drawing on insights from multiple sources and disciplines while helping to prevent any single perspective from dominating the interpretation. In order to strengthen its contribution, it is important to follow the principles of accurate, appropriate, and comprehensive reporting (Davies et al. 2023).

## 5 Conclusion

This study highlights the dynamic and multifactorial nature of SP beliefs, demonstrating how they evolve through the continuous interplay of lived experiences with symptoms, societal influences, and healthcare encounters. While social contexts and lived experiences shaped *informants'* beliefs, HPs appeared particularly influential, often reinforcing biomedical understandings. When these different sources of influence provided incongruent explanations, informants often experienced doubt and uncertainty, which at times undermined their confidence in existing explanatory frameworks. By applying Patient Journey Mapping, the study was able to capture moments of particular significance for informants, illustrating how SP beliefs develop and shift across time. Taken together, these findings not only demonstrate the value of Patient Journey Mapping as a methodological tool but also underscore the responsibility of HPs to communicate clearly and consistently, ensuring explanations match patients' health literacy, as their messages may exert lasting influence on individuals' understanding and management of SP.

## Author Contributions

C.B.F., M.F. and T.J. conceptualized the study and developed the interview guide. A.H. assisted with informant recruitment and booking of facilities. C.B.F. conducted and planned the interviews. M.F. transcribed recordings and provided design input for visualising the patient journey. C.B.F. and M.F. analysed the data and drafted the manuscript. All authors contributed to and approved the final manuscript.

## Ethics Statement

According to Danish legislation (Ministry of the Interior and Health 2024), § 14, committee act. 2, seeking approval from the ethical council when performing interview surveys when data is used anonymously is not necessary. Written consent was obtained from all informants, and they were offered to be sent a copy of the finished article after publication. Before the interview, informants were informed about the purpose of data collection, the procedures for handling and anonymising data in the article, and their right to withdraw from the study at any time.

## Conflicts of Interest

The authors declare no conflicts of interest.

## Supporting information


Supporting Information S1

## Data Availability

Research data are not publicly available due to privacy and ethical considerations, as the biographical nature of the interviews makes participants potentially identifiable even after anonymization.

## References

[msc70255-bib-0001] Alaiti, R. K. , J. Castro , H. Lee , et al. 2022. “What Are the Mechanisms of Action of Cognitive‐Behavioral, Mind‐Body, and Exercise‐Based Interventions for Pain and Disability in People With Chronic Primary Musculoskeletal Pain?: A Systematic Review of Mediation Studies From Randomized Controlled Trials.” Clinical Journal of Pain 38, no. 7: 502–509. 10.1097/AJP.0000000000001047. 35686580

[msc70255-bib-0002] Ben‐Tovim, D. I. , M. L. Dougherty , T. J. O’Connell , and K. M. McGrath . 2008. “Patient Journeys: The Process of Clinical Redesign.” Supplement, Medical Journal of Australia 188, no. S6: S14–S17. 10.5694/j.1326-5377.2008.tb01668.x. 18341470

[msc70255-bib-0003] Bunzli, S. , A. Smith , R. Schütze , and P. O’Sullivan . 2015. “Beliefs Underlying Pain‐Related Fear and How They Evolve: A Qualitative Investigation in People With Chronic Back Pain and High Pain‐Related Fear.” BMJ Open 5, no. 10: e008847. 10.1136/bmjopen-2015-008847. PMC4611881 26482773

[msc70255-bib-0004] Bunzli, S. , A. Smith , R. Schütze , I. Lin , and P. O’Sullivan . 2017. “Making Sense of Low Back Pain and Pain‐Related Fear.” Journal of Orthopaedic & Sports Physical Therapy 47, no. 9: 628–636. 10.2519/jospt.2017.7434. 28704621

[msc70255-bib-0005] Bunzli, S. , R. Watkins , A. Smith , R. Schütze , and P. O’Sullivan . 2013. “Lives on Hold: A Qualitative Synthesis Exploring the Experience of Chronic Low‐Back Pain.” Clinical Journal of Pain 29, no. 10: 907–916. 10.1097/AJP.0b013e31827a6dd8. 23370072

[msc70255-bib-0006] Caneiro, J. P. , S. Bunzli , and P. O’Sullivan . 2021. “Beliefs About the Body and Pain: The Critical Role in Musculoskeletal Pain Management.” Brazilian Journal of Physical Therapy 25, no. 1: 17–29. 10.1016/j.bjpt.2020.06.003. 32616375 PMC7817871

[msc70255-bib-0007] Cholewicki, J. , A. Breen , J. M. Popovich , et al. 2019. “Can Biomechanics Research Lead to More Effective Treatment of Low Back Pain? A Point‐Counterpoint Debate.” Journal of Orthopaedic & Sports Physical Therapy 49, no. 6: 425–436. 10.2519/jospt.2019.8825. 31092123 PMC7394249

[msc70255-bib-0008] Chou, L. , T. A. Ranger , W. Peiris , et al. 2018. “Patients’ Perceived Needs for Medical Services for Non‐Specific Low Back Pain: A Systematic Scoping Review.” PLoS One 13, no. 11: e0204885. 10.1371/journal.pone.0204885. 30408039 PMC6224057

[msc70255-bib-0009] Bonfim, I. D. S. , L. A. Corrêa , L. A. C. Nogueira , N. Meziat‐Filho , F. J. J. Reis , and R. S. de Almeida . 2021. “Your Spine Is So Worn Out’—The Influence of Clinical Diagnosis on Beliefs in Patients With Non‐Specific Chronic Low Back pain—A Qualitative Study.” Brazilian Journal of Physical Therapy 25, no. 6: 811–818. 10.1016/j.bjpt.2021.07.001. 34348864 PMC8721080

[msc70255-bib-0010] Darlow, B . 2016. “Beliefs About Back Pain: The Confluence of Client, Clinician and Community.” International Journal of Osteopathic Medicine 20: 53–61. 10.1016/j.ijosm.2016.01.005.

[msc70255-bib-0011] Darlow, B. , S. Dean , M. Perry , F. Mathieson , G. D. Baxter , and A. Dowell . 2015. “Easy to Harm, Hard to Heal: Patient Views About the Back.” Spine 40, no. 11: 842–850. 10.1097/BRS.0000000000000901. 25811262

[msc70255-bib-0012] Darlow, B. , A. Dowell , G. D. Baxter , F. Mathieson , M. Perry , and S. Dean . 2013. “The Enduring Impact of What Clinicians Say to People With Low Back Pain.” Annals of Family Medicine 11, no. 6: 527–534. 10.1370/afm.1518. 24218376 PMC3823723

[msc70255-bib-0013] Davies, E. L. , L. N. Bulto , A. Walsh , et al. 2023. “Reporting and Conducting Patient Journey Mapping Research in Healthcare: A Scoping Review.” Journal of Advanced Nursing 79, no. 1: 83–100. 10.1111/jan.15479. 36330555 PMC10099758

[msc70255-bib-0014] Deyo, R. A. , S. K. Mirza , J. A. Turner , and B. I. Martin . 2009. “Overtreating Chronic Back Pain: Time to Back Off?” Journal of the American Board of Family Medicine 22, no. 1: 62–68. 10.3122/jabfm.2009.01.080102. 19124635 PMC2729142

[msc70255-bib-0015] El‐Haddad, C. , A. Damodaran , H. Patrick McNeil , and W. Hu . 2018. “The Experience of Patients Admitted to Hospital With Acute Low Back Pain: A Qualitative Study.” International Journal of Rheumatic Diseases 21, no. 4: 796–803. 10.1111/1756-185X.12870. 27125577

[msc70255-bib-0016] Falsiroli Maistrello, L. , L. Zanconato , A. Palese , et al. 2022. “Perceptions and Experiences of Individuals With Neck Pain: A Systematic Critical Review of Qualitative Studies With Meta‐Summary and Meta‐Synthesis.” Physical Therapy 102, no. 8: pzac080. 10.1093/ptj/pzac080. 35708498 PMC9384136

[msc70255-bib-0017] Froud, R. , S. Patterson , S. Eldridge , et al. 2014. “A Systematic Review and Meta‐Synthesis of the Impact of Low Back Pain on People’s Lives.” BMC Musculoskeletal Disorders 15, no. 1: 50. 10.1186/1471-2474-15-50. 24559519 PMC3932512

[msc70255-bib-0018] GBD 2021 Low Back Pain Collaborators . 2023. “Global, Regional, and National Burden of Low Back Pain, 1990–2020, Its Attributable Risk Factors, and Projections to 2050: A Systematic Analysis of the Global Burden of Disease Study 2021.” Lancet Rheumatology 5, no. 6: e316–e329. 10.1016/S2665-9913(23)00098-X. 37273833 PMC10234592

[msc70255-bib-0019] Gulbrandsen, P. , H. B. Madsen , J. S. Benth , and E. Lærum . 2010. “Health Care Providers Communicate Less Well With Patients With Chronic Low Back pain—A Study of Encounters at a Back Pain Clinic in Denmark.” Pain 150, no. 3: 458–461. 10.1016/j.pain.2010.05.024. 20705213

[msc70255-bib-0020] Haldeman, S . 1990. “North American Spine Society: Failure of the Pathology Model to Predict Back Pain.” Spine 15, no. 7: 718–724. PMID: 2145646. 2145646

[msc70255-bib-0021] Hansen, A. , L. Morsø , M. J. Stochkendahl , et al. 2023. “Demographic and Clinical Characteristics of Patients With Low Back Pain in Primary and Secondary Care Settings in Southern Denmark.” Scandinavian Journal of Primary Health Care 41, no. 2: 152–159. 10.1080/02813432.2023.2196548. 37154804 PMC10193863

[msc70255-bib-0022] Hartvigsen, J. , M. J. Hancock , A. Kongsted , et al. 2018. “What Low Back Pain Is and Why We Need to Pay Attention.” Lancet 391, no. 10137: 2356–2367. 10.1016/S0140-6736(18)30480-X. 29573870

[msc70255-bib-0023] Hohenschurz‐Schmidt, D. , O. P. Thomson , G. Rossettini , et al. 2022. “Avoiding Nocebo and Other Undesirable Effects in Chiropractic, Osteopathy and Physiotherapy: An Invitation to Reflect.” Musculoskeletal Science & Practice 62: 102677. 10.1016/j.msksp.2022.102677. 36368170

[msc70255-bib-0024] Hoy, D. G. , M. Protani , R. De , and R. Buchbinder . 2010. “The Epidemiology of Neck Pain.” Best Practice & Research Clinical Rheumatology 24, no. 6: 783–792. 10.1016/j.berh.2011.01.019. 21665126

[msc70255-bib-0025] Kvale, S. , and S. Brinkmann . 2022. Interview: Det kvalitative forskningsinterview Som Håndværk. 3. edition Hans Reitzel.

[msc70255-bib-0026] Lederman, E . 2011. “The Fall of the Postural‐Structural‐Biomechanical Model in Manual and Physical Therapies: Exemplified by Lower Back Pain.” Journal of Bodywork and Movement Therapies 15, no. 2: 131–138. 10.1016/j.jbmt.2011.01.011. 21419349

[msc70255-bib-0027] Lin, I. B. , P. B. O’Sullivan , J. A. Coffin , D. B. Mak , S. Toussaint , and L. M. Straker . 2013. “Disabling Chronic Low Back Pain as an Iatrogenic Disorder: A Qualitative Study in Aboriginal Australians.” BMJ Open 3, no. 4: e002654. 10.1136/bmjopen-2013-002654. PMC3641505 23575999

[msc70255-bib-0028] Maher, C. , M. Underwood , and R. Buchbinder . 2017. “Non‐Specific Low Back Pain.” Lancet (London, England) 389, no. 10070: 736–747. 10.1016/S0140-6736(16)30970-9. 27745712

[msc70255-bib-0029] Maia, L. B. , J. P. Silva , M. B. Souza , N. Henschke , and V. C. Oliveira . 2021. “Popular Videos Related to Low Back Pain on YouTube^TM^ Do Not Reflect Current Clinical Guidelines: A Cross‐Sectional Study.” Brazilian Journal of Physical Therapy 25, no. 6: 803–810. 10.1016/j.bjpt.2021.06.009. 34332887 PMC8721063

[msc70255-bib-0030] Malterud, K . 2012. “Systematic Text Condensation: A Strategy for Qualitative Analysis.” Scandinavian Journal of Public Health 40, no. 8: 795–805. 10.1177/1403494812465030. 23221918

[msc70255-bib-0031] Malterud, K. , V. D. Siersma , and A. D. Guassora . 2016. “Sample Size in Qualitative Interview Studies: Guided by Information Power.” Qualitative Health Research 26, no. 13: 1753–1760. 10.1177/1049732315617444. 26613970

[msc70255-bib-0032] Ministry of the Interior and Health . 2024. “Bekendtgørelse af lov om videnskabsetisk behandling af sundhedsvidenskabelige forskningsprojekter og sundhedsdatavidenskabelige forskningsprojekter [Executive Order on the Act on Ethical Review of Health Research Projects and Health Data Science Research Projects] (Consolidated Act No. 1268 of 28 November 2024).” Retsinformation. https://www.retsinformation.dk/eli/lta/2024/1268.

[msc70255-bib-0033] Morlion, B. , G. Finco , D. Aldington , M. Überall , and R. Karra . 2021. “Severe Chronic Low Back Pain: Patient Journey From Onset of Symptoms to Strong Opioid Treatments in Europe.” Pain Management 11, no. 5: 595–602. 10.2217/pmt-2021-0009. 33847146

[msc70255-bib-0034] Morton, L. , M. de Bruin , M. Krajewska , D. Whibley , and G. J. Macfarlane . 2019. “Beliefs About Back Pain and Pain Management Behaviours, and Their Associations in the General Population: A Systematic Review.” European Journal of Pain 23, no. 1: 15–30. 10.1002/ejp.1285. 29984553 PMC6492285

[msc70255-bib-0035] Ng, W. , H. Slater , C. Starcevich , A. Wright , T. Mitchell , and D. Beales . 2021. “Barriers and Enablers Influencing Healthcare Professionals’ Adoption of a Biopsychosocial Approach to Musculoskeletal Pain: A Systematic Review and Qualitative Evidence Synthesis.” Pain 162, no. 8: 2154–2185. 10.1097/j.pain.0000000000002217. 33534357

[msc70255-bib-0036] Oosterhaven, J. , C. D. Pell , C. D. Schröder , et al. 2023. “Health Literacy and Pain Neuroscience Education in an Interdisciplinary Pain Management Programme: A Qualitative Study of Patient Perspectives.” PAIN Reports 8, no. 6: e1093. 10.1097/PR9.0000000000001093. 37868618 PMC10586826

[msc70255-bib-0037] O’Sullivan, P. , J. P. Caneiro , M. O’Keeffe , and K. O’Sullivan . 2016. “Unraveling the Complexity of Low Back Pain.” Journal of Orthopaedic & Sports Physical Therapy 46, no. 11: 932–937. 10.2519/jospt.2016.0609. 27802794

[msc70255-bib-0038] O’Sullivan, P. B. , L. M. Straker , A. Smith , M. Perry , and G. Kendall . 2008. “Carer Experience of Back Pain Is Associated With Adolescent Back Pain Experience Even When Controlling for Other Carer and Family Factors.” Clinical Journal of Pain 24, no. 3: 226–231. 10.1097/AJP.0b013e3181602131. 18287828

[msc70255-bib-0039] Petersen, L. , R. Birkelund , and B. Schiøttz‐Christensen . 2020. “Experiences and Challenges to Cross‐Sectoral Care Reported by Patients With Low Back Pain: A Qualitative Interview Study.” BMC Health Services Research 20, no. 1: 96. 10.1186/s12913-020-4952-x. 32028943 PMC7006064

[msc70255-bib-0040] Peterson, S. , N. Rainey , and K. Weible . 2022. “Who Writes This Stuff? Musculoskeletal Information Quality and Authorship of Popular Health Websites: A Systematic Review.” Musculoskeletal Science & Practice 60: 102563. 10.1016/j.msksp.2022.102563. 35453015

[msc70255-bib-0041] Ray, B. M. , A. Kovaleski , K. J. Kelleran , et al. 2022. “An Exploration of Low Back Pain Beliefs in a Northern America Based General Population.” Musculoskeletal Science & Practice 61: 102591. 10.1016/j.msksp.2022.102591. 35777261

[msc70255-bib-0042] Riipinen, P. , M. Holmes , S. Ogilvie , D. Newell , D. Byfield , and A. du Rose . 2022. “Patient’s Perception of Exercise for Management of Chronic Low Back Pain: A Qualitative Study Exercise for the Management of Low Back Pain.” Musculoskeletal Care 20, no. 4: 848–859. 10.1002/msc.1637. 35384268

[msc70255-bib-0043] Ryan, C. , and L. Roberts . 2019. ““Life on Hold”: The Lived Experience of Radicular Symptoms. A Qualitative, Interpretative Inquiry.” Musculoskeletal Science & Practice 39: 51–57. 10.1016/j.msksp.2018.11.005. 30500719

[msc70255-bib-0044] Schütze, R. , B. Liew , J. P. Caneiro , et al. 2025. “Mechanisms of Change in Cognitive Functional Therapy: A Longitudinal Mediation Analysis of the RESTORE Clinical Trial for Disabling Chronic Low Back Pain.” Behaviour Research and Therapy 193: 104853. 10.1016/j.brat.2025.104853. 40925074

[msc70255-bib-0045] Setchell, J. , N. Costa , M. Ferreira , J. Makovey , M. Nielsen , and P. W. Hodges . 2017. “Individuals’ Explanations for Their Persistent or Recurrent Low Back Pain: A Cross‐Sectional Survey.” BMC Musculoskeletal Disorders 18, no. 1: 466. 10.1186/s12891-017-1831-7. 29149847 PMC5693501

[msc70255-bib-0046] Singh, G. , C. Newton , K. O’Sullivan , A. Soundy , and N. R. Heneghan . 2018. “Exploring the Lived Experience and Chronic Low Back Pain Beliefs of English‐Speaking Punjabi and White British People: A Qualitative Study Within the NHS.” BMJ Open 8, no. 2: e020108. 10.1136/bmjopen-2017-020108. PMC5829944 29440143

[msc70255-bib-0047] Snelgrove, S. , and C. Liossi . 2009. “An Interpretative Phenomenological Analysis of Living With Chronic Low Back Pain.” British Journal of Health Psychology 14, no. Pt 4: 735–749. 10.1348/135910709X402612. 19187575

[msc70255-bib-0048] Stenberg, G. , A. Fjellman‐Wiklund , and C. Ahlgren . 2014. “’I Am Afraid to Make the Damage Worse’—Fear of Engaging in Physical Activity Among Patients With Neck or Back pain—A Gender Perspective.” Scandinavian Journal of Caring Sciences 28, no. 1: 146–154. 10.1111/scs.12043. 23578006

[msc70255-bib-0049] Stilwell, P. , and K. Harman . 2017. “Contemporary Biopsychosocial Exercise Prescription for Chronic Low Back Pain: Questioning Core Stability Programs and Considering Context.” Journal of the Canadian Chiropractic Association 61, no. 1: 6–17. PMID: 28413219. 28413219 PMC5381485

[msc70255-bib-0050] Tong, A. , P. Sainsbury , and J. Craig . 2007. “Consolidated Criteria for Reporting Qualitative Research (COREQ): A 32‐Item Checklist for Interviews and Focus Groups.” International Journal for Quality in Health Care 19, no. 6: 349–357. 10.1093/intqhc/mzm042. 17872937

[msc70255-bib-0051] Toye, F. , K. Seers , E. Hannink , and K. Barker . 2017. “A Mega‐Ethnography of Eleven Qualitative Evidence Syntheses Exploring the Experience of Living With Chronic Non‐Malignant Pain.” BMC Medical Research Methodology 17, no. 1: 116. 10.1186/s12874-017-0392-7. 28764666 PMC5540410

[msc70255-bib-0052] Valenzuela‐Pascual, F. , E. García‐Martínez , F. Molina‐Luque , et al. 2021. “Patients’ and Primary Healthcare Professionals’ Perceptions Regarding Chronic Low Back Pain and Its Management in Spain: A Qualitative Study.” Disability & Rehabilitation 43, no. 18: 2568–2577. 10.1080/09638288.2019.1705923. 31868034

[msc70255-bib-0053] van der Noord, R. , R. R. Reezigt , D. Paap , H. R. Schiphorst Preuper , and M. F. Reneman . 2025. “Unhelpful Information About Low Back and Neck Pain on Physiotherapist’s Websites.” European Journal of Pain 29, no. 2: e4782. 10.1002/ejp.4782. 39791187 PMC11718599

[msc70255-bib-0054] Vaz, D. V. , P. Stilwell , S. Coninx , M. Low , and C. Liebenson . 2023. “Affordance‐Based Practice: An Ecological‐Enactive Approach to Chronic Musculoskeletal Pain Management.” Brazilian Journal of Physical Therapy 27, no. 5: 100554. 10.1016/j.bjpt.2023.100554. 37925996 PMC10632936

[msc70255-bib-0055] Wand, B. M. , A. G. Cashin , J. H. McAuley , M. K. Bagg , G. M. Orange , and G. L. Moseley . 2023. “The Fit‐for‐Purpose Model: Conceptualizing and Managing Chronic Nonspecific Low Back Pain as an Information Problem.” Physical Therapy 103, no. 2: pzac151. 10.1093/ptj/pzac151. 36317747

[msc70255-bib-0056] Williams, S. E. , J. H. Ford , and E. A. Kensinger . 2022. “The Power of Negative and Positive Episodic Memories.” Cognitive, Affective, & Behavioral Neuroscience 22, no. 5: 869–903. 10.3758/s13415-022-01013-z. PMC9196161 35701665

[msc70255-bib-0057] Zheluk, A. , J. Anderson , and S. Dineen‐Griffin . 2022. “Analysis of Acute Non‐Specific Back Pain Content on Tiktok: An Exploratory Study.” Cureus 14, no. 1: e21404. 10.7759/cureus.21404. 35198311 PMC8856647

[msc70255-bib-0058] Zusman, M . 2013. “Belief Reinforcement: One Reason Why Costs for Low Back Pain Have Not Decreased.” Journal of Multidisciplinary Healthcare 6: 197–204. 10.2147/JMDH.S44117. 23717046 PMC3663473

