# Supplementary material for: The Development of Spinal Pain Beliefs: Patient Journey Mapping From Onset of Symptoms to MRI: A Qualitative Interview Study
Source: Musculoskeletal Care. 2026 Aug 7;24(3):e70255. doi: 10.1002/msc.70255 (PMC13451550; doi:10.1002/msc.70255)
Supplement: Supplementary file 1 [file MSC-24-e70255-s001.docx]

**Semi-structured interview guide**

| Themes | Questions |
| --- | --- |
| Thoughts and beliefs | - Tell me how your pain started. - Where did you first seek help, and what happened? - What were your expectations for the visit to…? (e.g., doctor, physiotherapist, chiropractor, etc.) - What thoughts/considerations/concerns did you have and had earlier regarding your spinal pain? - What challenges have there been in your course of treatment? |
| Spinal pain explanations | - What did you think was the cause of your spinal pain? - What explanations have you been given regarding your spinal pain? - Where did/do you search for information regarding your spinal pain? - What knowledge did you have about spinal pain before you experienced pain yourself? - How did you know that you needed help for your spinal pain? - What do you think is the cause of your spinal pain today? - What do you think about the fact that some people are unable to get a specific diagnosis/cause that can explain their pain? - Who had the most influence on what you think about your spinal pain? |
| Expectations regarding prognosis | - What expectations did you have for the future regarding your spinal pain? - What expectations do you have for your course treatment at the Spine Center? - What will it mean to you to get X investigation? (e.g., the contribution to the treatment/management of spinal pain) - When did you first think you needed a referral to the Spine Center? - What expectations do you have for the future regarding your spinal pain? |
